# A randomized controlled trial of a 5‐year marriage checkup booster session for a subsample of responder couples

**DOI:** 10.1111/jmft.12601

**Published:** 2022-09-24

**Authors:** Astrid B. Leth‐Nissen, Hanne N. Fentz, Gertraud Stadler, Tea L. Trillingsgaard

**Affiliations:** ^1^ The Department of Psychology and Behavioural Sciences Aarhus University Aarhus Denmark; ^2^ Charité—Universitätsmedizin Berlin, CC1 Health & Human Sciences Gender in Medicine Berlin Germany

**Keywords:** booster effects, brief couple interventions, intimacy, longitudinal design, marriage checkup, relationship satisfaction, responder sample

## Abstract

This study examined maintenance and booster effects of a brief couple intervention, the Marriage Checkup (MC), across 5 years. A subsample of 63 couples who benefitted from two previous MCs (responder couples), were randomly assigned to a third MC or control. Before randomization (at 4‐years‐9‐months), the responder sample had maintained small to medium effects on two measures of relationship functioning. After randomization, we found no significant between‐group effects. Yet, within‐group analyses revealed that while control couples showed flat trajectories in all outcomes after the 4‐year‐9‐months baseline, couples receiving a third MC (at Year 5) reported small to medium improvements in three measures of relationship functioning and maintained follow‐up effect in one measure. Findings indicate that couples who initially improved from the MC can maintain some of their improvements over long periods. The potential of boosting such improvements with recurrent MCs is a relevant target for further investigation in larger samples.

## INTRODUCTION

Couple distress and conflicts are common phenomena and have consistently been linked with reduced physical and mental health (e.g., Robles et al., 2014; Whisman, 2007). Couple therapy is effective (Roddy et al., 2020), yet most couples do not seek this type of help, or they delay help‐seeking until problems have accumulated to a point where the efficacy of couple therapy is reduced (for review of this point see Stewart et al., 2016). Barriers for seeking professional help include social stigma, lack of money and time, privacy concerns, and lack of adequate services (e.g., Williamson et al., 2019). These barriers challenge the reach and timely dissemination of interventions crucial for preventing and treating couple distress.

The marriage checkup (MC; Cordova, 2014) is a brief, low‐cost, and empirically supported couple intervention designed to lower the barriers for seeking professional help. Equivalent to other checkups (e.g., physical health), the MC provides regular contacts with a professional and is designed to bridge the gap between universal prevention and indicated treatment for couples (Cordova et al., 2014). Promoting both *“relationship health maintenance, early problem detection, and early intervention”* (Cordova et al., 2014; p. 593) the MC has been found to attract couples across the continuum from happy to severely distressed and to reach couples who never previously have sought professional help for their relationship (Cordova et al., 2005; Morrill et al., 2011). Likewise, couples perceive the MC as more accessible and less intimidating than traditional therapy (Morrill et al., 2011). When imported to Denmark in 2016, the MC was adapted to private practice, and the Danish format consists of two joint 90 min sessions (assessment and feedback; Trillingsgaard et al., 2017). The MC aims to foster intimacy in couples and builds on methods from integrative behavioral couple therapy (Christensen et al., 2020), motivational interviewing, and relationship health education (Cordova, 2014). Though manualized, the therapist flexibly tailors the MC to the couple's unique strengths and concerns and identifies the most adequate plan for subsequent caretaking of the specific relationship.

Randomized control trials in the United States and in Denmark have found small to medium effects on relationship functioning as well as on individual depression symptoms of two annual MCs across 1 or 2 years (e.g., Cordova et al., 2014; Gray et al., 2020; Trillingsgaard et al., 2016). The MC has been successfully adapted to different populations including military couples (Cordova et al., 2017), perinatal couples (Darling et al., 2021), low‐income at‐risk couples (Gordon et al., 2019), Korean couples (Lee & Kwon, 2018), lesbian couples (Minten & Dykeman, 2019), transgender couples (pilot study by Minten & Dykeman, 2021), and couples who disagreed on relationship concerns (Reyes et al., 2020). Taken together, the MC is classified as a well‐established intervention meeting the criteria for the highest level of evidence outlined by Wittenborn and Holtrop (2021; adapted from Southam‐Gerow & Prinstein, 2014), as also concluded by Doss et al. (2021).

In terms of improving relationship health, results of the previous RCTs (Cordova et al., 2014; Trillingsgaard et al., 2016) visualized a trajectory of change shaped as a *climbing M*; small positive increases after the first MC, then a small decrease followed by small to medium increases after the second MC. Most measures even indicated an *anticipation effect* (Cordova, 2014), with increases in relationship functioning *prior* to the second MC. Some decreases were found at follow‐up, but most measures maintained significant improvements (Cordova et al., 2014; Trillingsgaard et al., 2016). These findings reflect the assumption that the MC (as any checkup model in its definition) promotes healthy relationship maintenance through brief but regular care (e.g., Cordova et al., 2014). Inherent in this assumption is that longitudinal effects of the MC depend on recurring booster sessions over time. To date, no study has followed couples through more than two annual MCs and beyond 2 years which leaves the longitudinal effects of the MC untested.

### A checkup model for whom and when: Selecting a sample of responder couples

A well‐established finding from longitudinal studies of couples is that their average level of relationships satisfaction generally declines over time (see e.g., the systematic review and meta‐analysis by Bühler et al., 2021). This consistent average decline could lead one to argue that all couples are relevant targets for preventive interventions. However, recent research using latent class approaches shows substantial heterogeneity in trajectories of relationship satisfaction so that a *minority* of couples experience steep decline over time, while the majority (50%−90%) of couples show insignificant or minimal decline (for a review of the past two decades of research in the field see e.g., Fentz et al., 2022; Karney & Bradbury, 2020; Proulx et al., 2017). Furthermore, couples' low initial satisfaction level is predictive of deterioration, while initially satisfied couples usually stay satisfied over time (Fentz et al., 2022; Karney & Bradbury, 2020). Previous RCTs of the MC recruited couples across the full spectrum of relationship satisfaction (Cordova et al., 2014; Trillingsgaard et al., 2016). Although these studies found small to medium *average* effects on relationship functioning from receiving two MCs, these effects cover a considerable heterogeneity with only 10.2%−33.3% of the couples reaching a reliable change (depending on measurement and timepoint, based on the Reliable Change Index; Jacobson & Truax, 1991). Taken together, findings imply that not all couples are equally relevant recipients of preventive interventions. Couples who did not meet the criteria for a reliable change may be couples who (a) received the MC at a happy time of their relationship with little room for improvement and a prognosis of stability, (b) were severely distressed and in need of a different type of help (e.g., couple therapy, individual therapy), or (c) received the MC too late in terms of relational damage and lack of commitment to invest in the relationship. Outside a research setting serving free recurring MCs for all couples, these types of nonresponder couples would probably be served a first checkup and then referred to either a more intensive service (e.g., couple therapy or divorce counseling) or a less intensive service (self‐directed maintenance of the relationship). Each type of nonresponder couples would be less optimal candidates for recurrent checkups as the timing and dose of an additional MC might not be appropriate. From a cost‐effective perspective, the most relevant recipients of recurrent MCs are responder couples for whom the first MC was an adequate and beneficial dose of care.

### Aim

This current RCT aimed to test if couples who have benefitted from two MCs can maintain and boost their relationship functioning with a third MC provided at Year 5. Using a sample of responder couples, we target this aim with three main analyses of (1) the long‐term maintained effects of the two previous MCs prior to the current baseline, (2) the between‐ and within‐group booster effects of the third MC using the current baseline, and, finally, (3) the between‐ and within‐group booster effects of the third MC using the original baseline in the previous RCT in order to graph the outcome variables across the full study period of 5 years and 3 months.

## METHODS

### Design

This RCT extends a previous RCT (Trillingsgaard et al., 2016) in which 233 couples were randomized to an intervention group receiving two MCs scheduled 1 year apart or a control group receiving movie tickets and a feedback report. Details and results are described in Trillingsgaard et al. (2016).

In the current study, we randomized couples, who benefitted from the two previous MCs (referred to as responder couples), to either a new intervention condition receiving a third MC (*n* = 32) or a control condition receiving no further intervention (*n* = 31). This re‐randomization took place 4 years and 9 months after the original baseline. The intervention was conducted by one of five trained psychologists at one of two sites, the private clinic of Center for Family Development in Copenhagen, or the university clinic at Aarhus University. Data were collected through online surveys at the 4‐year‐9‐months baseline (Week 0), prior to the MC (Week 16, intervention group only), 2 weeks after (Week 18), and at follow‐up (Week 28).

### Recruitment and inclusion procedure

**Figure 1:**
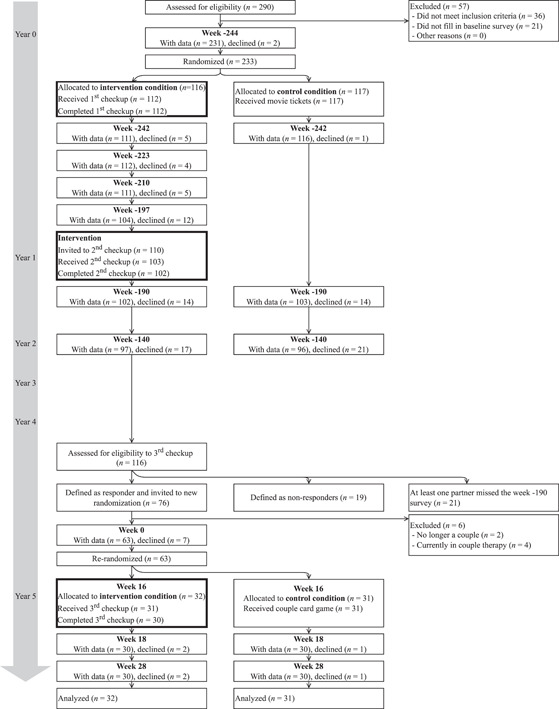
Consort diagram. *n*, number of couples

Couples were eligible for the current study if they were responders defined by the following criteria: Both partners had provided data at the original baseline (Week −244, see Figure 1) and at 2 weeks after the second MC (Week −190), and at least one partner showed a positive reliable change ($1.96\times \sqrt{{2\times \mathrm{Standard}\text{Error}}^{2}}$; Jacobson & Truax, 1991) from the original baseline (Week −244) to after the second MC (Week −190) on at least one of the four outcome variables. A medium size of effect was reasonable to expect from a sample of previous responders. When accounting for an attrition rate of 20%, a power analysis indicated that a sample of 72 couples would be needed to detect a between‐group difference with a medium effect size (Cohen's *d* of 0.65) with a power of 0.8. We classified all couples in which both partners had answered at both timepoints (*n* = 95 couples) into responders versus nonresponders, resulting in 65 eligible couples. To allow for attrition, we lowered the criterion for a reliable change by 20%, resulting in a total sample of 76 couples (80% of the 95 couples). Invitations were sent to each partner by e‐mail and included a personal login to the online registration and consent form. We enrolled couples across two waves, May and August 2018.

Figure 1 illustrates the 5‐year flow of the participants beginning with the original RCT and continuing through the current RCT. Prior to the current baseline survey (Week 0), 6 out of the 76 couples were excluded because they were no longer a couples or were currently receiving couple therapy, and 7 couples declined the invitation expressing that they were satisfied with their relationship and therefore in no need for intervention. When both partners had answered the current baseline survey (Week 0, *N* = 63 couples), they were immediately rerandomized using sequentially numbered, opaque, sealed envelopes (Doig & Simpson, 2005). No monetary incentives were given to participants but couples in the control group were compensated at Week 16 with a gift consisting of a card game for couples. All 63 couples were included and analyzed within an intention to treat (ITT) approach. All study procedures complied with standards from the regional ethical committee, and the study was approved by the Danish Data Protection Agency.

### Measures

#### Relationship satisfaction

Global relationship satisfaction was measured using the marital satisfaction inventory—brief (MSI; 10 items; Whisman et al., 2009) and the couple satisfaction index (CSI; 16 items; Funk & Rogge, 2007). On CSI, respondents rate their level of satisfaction on 6‐ or 7‐point Likert scales with a possible sum score ranging from 0 to 81 and a higher score indicating higher satisfaction. Item samples of the CSI include *“My relationship with my partner makes me happy”* (rated from 0 = *not at all true*, to 5 = *completely true*) and *“Please indicate the degree of happiness, all things considered, of your relationship”* (rated from 0 = *Extremely unhappy*, to 6 = *Perfect*). We defined a couple as experiencing relational distress if at least one partner scored below 51.5 on the CSI (Funk & Rogge, 2007). The CSI showed high internal reliability in the current study (Cronbach's *α* at Week −244 = 0.97; *N* = 462 participants). The MSI was originally developed to screen for relationship discord and is composed of 10 items deriving from five different scales of the MSI‐Revised (Whisman et al., 2009). Items are rated binary (as *Yes* or *No*) with a possible sum score ranging from 0 to 10 and a higher score indicates less distress. Item samples of the MSI include *“There are some serious difficulties in our relationship”* and *“Our sexual relationship is entirely satisfactory*.*”* The Cronbach's *α* of the MSI in the current study was 0.66 (Week −244) which is considered acceptable given that the MSI measures different relationship functioning domains. Though deriving from different scales, the 10 items of the MSI have been found to measure the same underlying latent construct of relationship distress (Balderrama‐Durbin et al., 2015).

#### Intimacy

Intimacy was measured using the intimacy safety questionnaire (ISQ; 27 items; Cordova et al., 2005). Respondents rate their feeling of intimacy with their partner on 5‐point Likert scales, and a mean score is calculated ranging from 0 to 4 with a higher score indicating more intimacy. Item samples include *“I feel comfortable telling my partner when I'm feeling sad”* (0 = *Never*, 4 = *Always*) and *“I feel like I have to watch what I do or say around my partner”* (0 = *Always*, 4 = *Never*). The ISQ showed high internal reliability in the current study (Cronbach's *α* = 0.90 at Week −244).

#### Responsive attention

The participant's perception of their partner's daily provision of responsive attention was measured with the responsive attention scale (RAS; 12 items; Trillingsgaard & Fentz, 2016, ^1^). Items are rated on 5‐point Likert scales with a possible sum score ranging from 12 to 60 and a higher score indicating higher perceived responsive attention. Item samples include *“I receive a warm welcome when we meet at the end of the day”* and *“If I tell my partner about my day, he or she listens with interest”* which are rated from 1 = *Very rarely* to 5 = *Very often*. The Cronbach's *α* of the RAS was acceptable (*α* = 0.81; Week −244).

#### Therapist manual adherence

All MC sessions were videotaped, and manual adherence was coded on a random sample of 20% of the tapes by two independent raters following the procedure of Trillingsgaard et al. (2016). Therapist behavior was rated from 0 (*Did not adhere to manual)* to 5 (*Completely adhered to manual*) on 14 elements during the assessment and feedback sessions of the MC.

The average adherence rating was 4.77 (range from 3.88 to 5.00), indicating that therapists overall adhered to the MC manual. The interrater reliability was good as coders agreed within one level of the scale in 89.3% of their ratings.

### Data analyses

Data were analyzed in SPSS 27. Data set can be obtained from the last author upon request. We tested if the current intervention group (receiving a third MC) and control group differed on sociodemographic characteristics at the original baseline (Week −244) using Pearson's *χ* ^2^ tests for categorical variables and MANOVA for continuous variables. We also included two other groups in this comparison; couples classified as nonresponders (*n* = 19) and the original control group from the previous RCT (Trillingsgaard et al., 2016) who had never received any MC (*n* = 117; see Table 1). All other analyses in this study exclusively included the current responder sample (63 couples). To investigate the long‐term maintained effects from the original RCT baseline (Week −244) to the current baseline (Week 0) we conducted paired *t*‐tests on the pooled responder sample.

**Table 1:** Characteristics of participants (*N* = 199 couples) at original baseline Week −244

Baseline characteristic (Week −244)	Current RCT		For comparisons	
	Intervention (3 MCs, *n* = 32)	Control (2 MCs, *n* = 31)	Nonresponders (2 MCs, *n* = 19)	Original control (0 MC, *n* = 117)
Age				
Women, mean (*SD*)	38.77 (5.71)	35.38 (5.57)	36.78 (6.25)	37.86 (6.93)
Men, mean (*SD*)	40.43 (5.54)	36.99 (5.28)	37.77 (7.05)	39.57 (6.70)
Education of bachelor degree or above^a^, *n* (%)	51 (79.7)	52 (83.9)	34 (89.5)	203 (86.8)
Married, *n* (%)	29 (90.6)	25 (80.6)	14 (73.7)	92 (78.6)
Length of relationship (years), mean (*SD*)	12.94 (6.00)	12.45 (5.13)	12.37 (7.27)	11.74 (5.57)
Dual employment, *n* (%)	28 (87.5)	22 (71.0)	10 (52.6)	87 (74.4)
Born in Denmark^a^, *n* (%)	59 (92.9)	62 (100)	34 (91.9)	211 (93.4)
Study site				
Copenhagen, *n* (%)	15 (46.9)	16 (51.6)	9 (47.4)	55 (47.0)
Aarhus, *n* (%)	17 (53.1)	15 (48.4)	10 (52.6)	62 (53.0)

Abbreviation: MC, marriage checkup.

^a^ Calculated on individual rather than couple level.

To evaluate the treatment effects of the third MC, we used a dyadic score model following recommendations by Iida et al. (2018) using partner means ([female partner + male partner]/2) and partner differences ([female partner – male partner]/2) in relationship outcome variables. We used this dyadic score model to account for the considerable shared variance in the outcome variables, for example, the correlations between partners in baseline scores (at Week 0, *r*s = 0.33−0.49) and in change scores (from Week 0 to 28: *r*s = 0.22−0.33). We carefully tested for gender differences and effects in partner means and in partner differences. Although male partners were generally more satisfied than female partners (see Figure A1 in the appendix), we found effects only in partner means and not in partner differences. Thus, we report the more parsimonious model focusing on partner means. While the trajectories of change in the control group fitted a linear model, the trajectories among couples in the intervention group changed direction. To allow for these breaks, we built a factorial multilevel model with a random intercept and couple as clustering variable where each timepoint was added as a dummy coded (0/1) factor. We tested the between‐group difference in change and the within‐group change in two models. First, we modeled change in the current RCT as change from the current baseline (Week 0) to subsequent timepoints at Week 16 (only for the intervention group), 18, and 28 respectively. Second, we modeled change across all 5 years using the original RCT baseline (Week −244) as the reference and estimating change to after each of the three MCs (Week −242, −190, and 18), and to the 5‐year‐3‐months follow‐up (Week 28). Reporting of the within‐group effects was included because the sample is relatively small and because both groups originate from the same condition (receiving two MCs) and constitute a relatively homogeneous sample of responder couples. We calculated Cohen's *d* effect sizes by dividing the estimated effect at each timepoint by the pooled standard deviation of the 63 couples at either the current baseline or the original baseline.

We were able to obtain data on 95.5% of the maximum possible number of observations across Week 0−28 (844 obtained observations out of 884 possible observations^2^).

As the MC was developed for couples across the spectrum of relationship satisfaction, we conducted sensitivity analyses for the influence of severely distressed couples on treatment effects, where we identified couples as outliers if they showed one or more “probable outlying” low score (*z* > 2.58 equaling the 1% most extreme cases; Field, 2018) on at least one of the four outcome variables at Week 0, 16, 18, or 28.

## RESULTS

The sociodemographic characteristics of the couples randomized to the intervention (third MC) or the control condition in the current study are described in Table 1. This table also outlines characteristics of the nonresponder couples and the original control group from the previous RCT for comparison. These four groups did not differ significantly (*p*s > 0.131) at the original baseline (Week −244) with the single exception of fewer dual employed couples among nonresponders compared to the current intervention group (*p* < 0.01, Cramer's *V* = 0.39). Means and standard deviations of the four outcome variables at all 11 timepoints for the four groups are reported in Table A1 in the appendix.

### Long‐term maintenance of effects from original RCT to current baseline

The current responder couples (*N* = 63) had maintained small to medium long‐term effects of the two previous MCs across the 4 years and 9 months from their original baseline level (Week −244) to the current baseline (Week 0) on MSI (*d* = 0.51, *p* < 0.001) and ISQ (*d* = 0.39, *p* = 0.003). The RAS trended toward a significant maintained effect (*d* = 0.24, *p* = 0.058), while no effect was maintained for the CSI (*d* = 0.00, *p* = 0.990). At the current baseline, 22 of the 63 couples (34.9%) had at least one partner reporting relational distress.

### Booster effects of the third MC across 6 months

**Figure 2:**
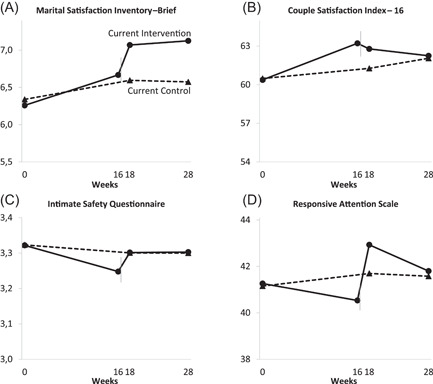
Outcome trajectories across Week 0−28 for couples in the current intervention group (receiving their third MC at Week 16) and control group. Three outlying couples omitted. *Y*‐axes are sized to one standard deviation. The time of the third MC is marked with thin vertical lines. MC, marriage checkup

**Figure A2:**
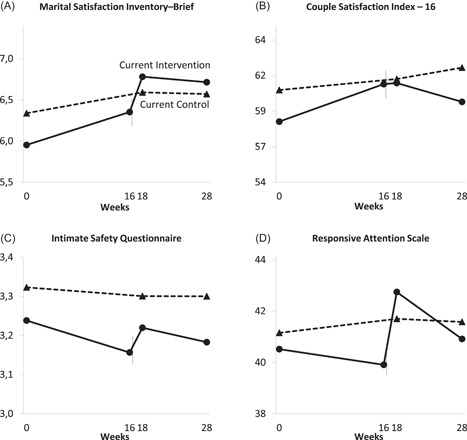
Outcome trajectories across Week 0−28 in the ITT intervention and control group. *Y*‐axes are sized to one standard deviation. The time of the MC is marked with thin vertical lines. ITT, intention to treat; MC, marriage checkup.

Results of the third MC (completed by 30 couples) are presented in Table 2. No significant baseline differences (at Week 0) were seen between the current intervention and control group in any of the outcome variables (*p*s > 0.367), as expected following random assignment. A possible source of bias was found in that three couples in the intervention group, but none in the control group, were identified with outlying low scores (indicating high level of distress). Sensitivity analyses revealed that our ITT results were not considerably changed by omitting the three outlying (highly distressed) couples. Still, to address these potential outliers, we visualized trajectories both with (Figure A2_A−D_) and without them (Figure 2_A−D_). Between‐group differences in change from baseline (Week 0) to after the third MC (Week 18) and to follow‐up (Week 28) were nonsignificant on all four outcomes, with a trend for a stronger increase on the MSI in the intervention group than the control group at Week 18 (*d* = 0.33, *p* = 0.061) and Week 28 (*d* = 0.31, *p* = 0.087). Within‐group analyses revealed that couples in the current control group showed statistically flat trajectories from the current baseline (Week 0) through the subsequent time points on all four outcomes (*p*s > 0.148). In the intervention group, couples showed different trajectories on each outcome: For global measures of relationship satisfaction, couples showed a trending, small anticipation effect on the MSI prior to the third MC (*d* = 0.23, *p* = 0.060) that increased to a significant small‐to‐medium effect after the MC (*d* = 0.48, *p* < 0.001) and was maintained at the 3‐months follow‐up (*d* = 0.44, *p* < 0.001). On the CSI, couples showed a small anticipation effect (*d* = 0.24, *p* = 0.012), which was maintained right after the MC (*d* = 0.25, *p* = 0.011), but had eroded at follow‐up. For intimacy, couples dropped prior to the MC with a small negative effect size (*d* = −0.24, *p* = 0.023). After the MC, the slight increase in intimacy was nonsignificant (*p* = 0.609). Couples did not change significantly on responsive attention prior to the MC but spiked after the MC with a small effect (*d* = 0.34, *p* = 0.014) that had eroded at follow‐up.

**Table 2:** Multilevel estimates of effects, effects sizes, and 95% confidence intervals across Week 0−28 (ITT)

Variable	Estimate	*SE*	*p*	*d* [CI]
**Marital satisfaction inventory–brief**				
Within‐group changes				
Intervention group				
Intercept at Week 0	**5.95**	0.33	<0.001	
Week 0 → 16	0.40	0.21	0.060	0.23 [−0.02 to 0.82]
Week 0 → 18	**0.83**	0.21	<0.001	**0.48** [0.41−1.25]
Week 0 → 28	**0.76**	0.21	<0.001	**0.44** [0.34−1.18]
Control group				
Intercept at Week 0	**6.34**	0.33	<0.001	
Week 0 → 18	0.25	0.22	0.246	0.15 [−0.18 to 0.69]
Week 0 → 28	0.23	0.22	0.291	0.13 [−0.20 to 0.67]
Between‐group differences (intervention‐control group)				
Group dif. at Week 0	−0.39	0.47	0.410	−0.22 [−1.31 to 0.54]
Group dif. in change Week 0 → 18	0.58	0.30	0.061	0.33 [−0.03 to 1.18]
Group dif. in change Week 0 → 28	0.53	0.31	0.087	0.31 [−0.08 to 1.14]
**Couples satisfaction index–16**				
Within‐group changes				
Intervention group				
Intercept at Week 0	**58.27**	1.93	<0.001	
0 → 16	**2.64**	1.04	0.012	**0.24** [0.59−4.69]
0 → 18	**2.71**	1.05	0.011	**0.25** [0.64−4.78]
0 → 28	1.39	1.05	0.186	0.13 [−0.68 to 3.46]
Control group				
Intercept at Week 0	**60.48**	1.96	<0.001	
Week 0 → 18	0.78	1.07	0.465	0.07 [−1.33 to 2.89]
Week 0 → 28	1.57	1.08	0.148	0.15 [−0.56 to 3.71]
Between‐group differences (intervention‐control group)				
Group dif. at Week 0	−2.22	2.75	0.422	−0.20 [−7.69 to 3.26]
Group dif. in change Week 0 → 18	1.93	1.49	0.199	0.18 [−1.02 to 4.88]
Group dif. in change Week 0 → 28	−0.18	1.50	0.906	−0.02 [−3.15 to 2.79]
**Intimate safety questionnaire**				
Within‐group changes				
Intervention group				
Intercept at Week 0	**3.24**	0.07	<0.001	
0 → 16	−**0.08**	0.04	0.023	−**0.24** [−0.15 to −0.01]
0 → 18	−0.02	0.04	0.609	−0.05 [−0.09 to 0.05]
0 → 28	−0.06	0.04	0.125	−0.16 [−0.13 to 0.02]
Control group				
Intercept at Week 0	**3.32**	0.07	<0.001	
Week 0 → 18	−0.02	0.04	0.538	−0.07 [−0.09 to 0.05]
Week 0 → 28	−0.02	0.04	0.539	−0.07 [−0.10 to 0.05]
Between‐group differences (intervention‐control group)				
Group dif at Week 0	−0.08	0.09	0.367	−0.25 [−0.27 to 0.10]
Group dif. in change Week 0 → 18	0.00	0.05	0.936	0.01 [−0.10 to 0.11]
Group dif. in change Week 0 → 28	−0.03	0.05	0.527	−0.10 [−0.13 to 0.07]
**Responsive attention scale**				
Within‐group changes				
Intervention group				
Intercept at Week 0	**40.72**	1.10	<0.001	
0 → 16	−0.61	0.88	0.492	−0.09 [−2.35 to 1.13]
0 → 18	**2.23**	0.89	0.014	**0.34** [0.47−3.99]
0 → 28	0.40	0.89	0.657	0.06 [−1.37 to 2.16]
Control group				
Intercept at Week 0	**41.35**	1.12	<0.001	
Week 0 → 18	0.55	0.91	0.546	0.08 [−1.24 to 2.34]
Week 0 → 28	0.42	0.92	0.644	0.06 [−1.39 to 2.24]
Between‐group differences (intervention‐control group)				
Group dif. at Week 0	−0.64	1.57	0.686	−0.10 [−3.75 to 2.48]
Group dif. in change Week 0 → 18	1.68	1.27	0.188	0.26 [−0.83 to 4.19]
Group dif. in change Week 0 → 28	−0.03	1.28	0.982	−0.00 [−2.56 to 2.50]

*Note*: The marriage checkup was delivered at Week 16. Significant (*p* < 0.05) estimated values and effect sizes are bolded.

Abbreviations: CI, 95% confidence interval; *d*, Cohen's *d*; dif., difference; ITT, intention to treat.

### Maintained and booster effects of three MCs across 5 years

**Figure 3:**
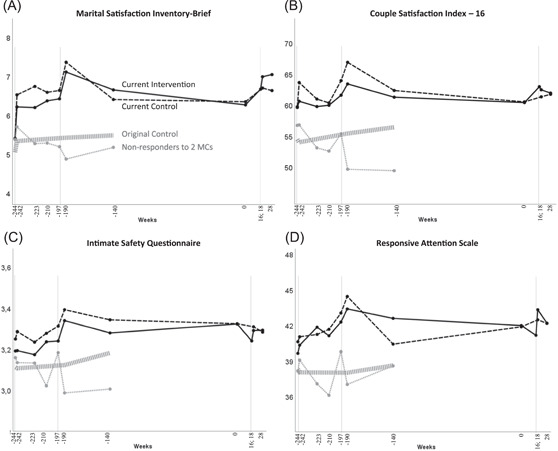
Outcome trajectories across 5 years and 3 months in the current intervention and control group, the nonresponder group, and the original control group (who did not receive any MC). Three outlying couples omitted. Graphs are based on the raw partner means since not all groups and timepoints were included in the current models. *Y*‐axes are sized to two standard deviations. The times of each MC are marked with thin vertical lines. MC, marriage checkup.

**Figure A3:**
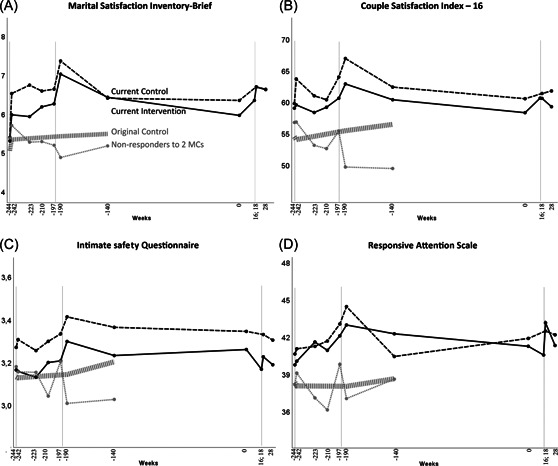
Outcome trajectories across 5 years and 3 months in the current ITT intervention and control group, the nonresponder group, and the original control group (who did not receive any MC). Graphs are based on the raw partner means since not all groups and timepoints were included in the current models. *Y*‐axes are sized to two standard deviations. The times each MC are marked with thin vertical lines.  ITT, intention to treat; MC, marriage checkup.

Effects in our responder couples of each of the three MCs (two received in the original RCT and one received in the current RCT) are presented in Table 3 and Figure 3_A−D_ (corresponding ITT graphs in Figure A3_A−D_). Beside the trajectories of the two current groups of responder couples, we also graphed the trajectories of the nonresponder couples and the original control couples from the previous RCT (who did not receive any MC) in Figure 3_A−D_ and Figure A3_A−D_. Between‐group differences in change were nonsignificant from the original baseline (Week −244) to after the third MC and to the 5‐year‐3‐months follow‐up in all outcome measures (*p*s > 0.113). Within‐group analyses revealed that when couples in the current intervention group had completed their third MC, they showed a large within‐group effect at Week 18 on MSI (*d* = 0.89, *p* < 0.001) compared to their original baseline (Week −244), a medium effect on responsive attention (*d* = 0.60, *p* < 0.001), and a trending, small effect on intimacy (*d* = 0.27, *p* = 0.094), while the effect on CSI was nonsignificant (*p* = 0.361). In comparison, the current control group had maintained a large effect on MSI (*d* = 0.75, *p* < 0.001) and a small effect on responsive attention (*d* = 0.31, *p* = 0.049), while the effects on CSI and ISQ were nonsignificant (*p*s > 0.320). At the 5‐year‐3‐months follow‐up, couples in the current intervention group had maintained a large effect on the MSI (*d* = 0.85, *p* < 0.001) and a trending, small effect on responsive attention (*d* = 0.29, *p* = 0.061), while they had not maintained effects on CSI and ISQ (*p*s > 0.386). The same follow‐up pattern was found in the current control group (MSI: *d* = 0.77, *p* < 0.001; RAS: *d* = 0.27, *p* = 0.086; CSI: *p* = 0.225; ISQ: *p* = 0.476).

**Table 3:** Multilevel estimates of effects, effects sizes, and 95% confidence intervals across 5 years from original baseline (Week −244) to post the 1st, 2nd, and 3rd MC, and to follow‐up (*N* = 63 responder couples)

Variable	Estimate	*SE*	*p*	*d* [CI]
**Marital satisfaction inventory–brief**				
Within‐group changes				
Intervention group				
Intercept at baseline (Week −244)	**5.30**	0.31	0.000	
Baseline → post 1st MC (Week −242)	**0.67**	0.29	0.023	**0.43** [0.09−1.25]
Baseline → post 2nd MC (Week −190)	**1.72**	0.29	0.000	**1.09** [1.14−2.30]
Baseline → post 3rd MC (Week 18)	**1.40**	0.30	0.000	**0.89** [0.81−1.99]
Baseline → follow‐up (Week 28)	**1.33**	0.30	0.000	**0.85** [0.74−1.92]
Control group				
Intercept at baseline (Week −244)	**5.39**	0.31	0.000	
Baseline → post 1st MC (Week −242)	**1.13**	0.30	0.000	**0.72** [0.54−1.72]
Baseline → post 2nd MC (Week −190)	**1.97**	0.30	0.000	**1.25** [1.38−2.55]
Baseline → post 3rd MC (Week 18)	**1.17**	0.30	0.000	**0.75** [0.57−1.77]
Baseline → follow‐up (Week 28)	**1.21**	0.31	0.000	**0.77** [0.61−1.82]
Between‐group differences (intervention‐control group)				
Group dif. at baseline	−0.09	0.44	0.838	−0.06 [−0.96 to 0.78]
Group dif. in change after 1st MC	−0.46	0.42	0.275	−0.29 [−1.28 to 0.37]
Group dif. in change after 2nd MC	−0.25	0.42	0.552	−0.16 [−1.07 to 0.57]
Group dif. in change after 3rd MC	0.23	0.43	0.595	0.14 [−0.61 to 1.07]
Group dif. in change at follow‐up	0.12	0.43	0.779	0.08 [−0.72 to 0.97]
**Couples satisfaction index–16**				
Within‐group changes				
Intervention group				
Intercept at baseline (Week −244)	**58.98**	1.71	0.000	
Baseline → post 1st MC (Week −242)	0.52	1.49	0.730	0.05 [−2.43 to 3.46]
Baseline → post 2nd MC (Week −190)	**3.84**	1.49	0.011	**0.39** [0.90−6.79]
Baseline → post 3rd MC (Week 18)	1.40	1.53	0.361	0.14 [−1.61 to 4.40]
Baseline → follow‐up (Week 28)	0.08	1.53	0.959	0.01 [−2.93 to 3.08]
Control group				
Intercept at baseline (Week −244)	**59.71**	1.74	0.000	
Baseline → post 1st MC (Week −242)	**3.90**	1.52	0.011	**0.40** [0.91−6.89]
Baseline → post 2nd MC (Week −190)	**7.16**	1.52	0.000	**0.73** [4.17−10.15]
Baseline → post 3rd MC (Week 18)	0.96	1.55	0.534	0.10 [−2.09 to 4.02]
Baseline → follow‐up (Week 28)	1.91	1.57	0.225	0.19 [‐1.18 to 4.99]
Between‐group differences (intervention‐control group)				
Group dif. at baseline	−0.73	2.44	0.767	−0.07 [−5.56 to 4.11]
Group dif. in change after 1st MC	−3.39	2.13	0.113	−0.34 [−7.58 to 0.81]
Group dif. in change after 2nd MC	−3.32	2.13	0.121	−0.34 [−7.51 to 0.88]
Group dif. in change after 3rd MC	0.43	2.17	0.843	0.04 [−3.85 to 4.71]
Group dif. in change at follow‐up	−1.83	2.19	0.404	−0.19 [−6.14 to 2.48]
**Intimate safety questionnaire**				
Within‐group changes				
Intervention group				
Intercept at baseline (Week −244)	**3.13**	0.06	0.000	
Baseline → post 1st MC (Week −242)	0.01	0.04	0.863	0.03 [−0.08 to 0.10]
Baseline → post 2nd MC (Week −190)	**0.15**	0.04	0.001	**0.53** [0.06−0.24]
Baseline → post 3rd MC (Week 18)	0.08	0.05	0.094	0.27 [−0.01 to 0.17]
Baseline → follow‐up (Week 28)	0.04	0.05	0.386	0.14 [−0.05 to 0.13]
Control group				
Intercept at baseline (Week −244)	**3.25**	0.06	0.000	
Baseline → post 1st MC (Week −242)	0.04	0.04	0.416	0.13 [−0.05 to 0.12]
Baseline → post 2nd MC (Week −190)	**0.14**	0.04	0.002	**0.51** [0.05−0.23]
Baseline → post 3rd MC (Week 18)	0.05	0.05	0.320	0.16 [−0.04 to 0.14]
Baselin → follow‐up (Week 28)	0.03	0.05	0.476	0.12 [−0.06 to 0.12]
Between‐group differences (intervention‐control group)				
Group dif. at baseline	−0.12	0.08	0.155	−0.43 [−0.29 to 0.05]
Group dif. in change after 1st MC	−0.03	0.06	0.649	−0.10 [−0.15 to 0.10]
Group dif. in change after 2nd MC	0.00	0.06	0.937	0.02 [−0.12 to 0.13]
Group dif. in change after 3rd MC	0.03	0.06	0.632	0.11 [−0.10 to 0.16]
Group dif. in change at follow‐up	0.01	0.06	0.921	0.02 [−0.12 to 0.13]
**Responsive attention scale**				
Within‐group changes				
Intervention group				
Intercept at baseline (Week −244)	**39.22**	0.99	0.000	
Baseline → post 1st MC (Week −242)	0.30	0.88	0.736	0.05 [−1.44 to 2.03]
Baseline → post 2nd MC (Week −190)	**3.22**	0.88	0.000	**0.55** [1.49−4.95]
Baseline → post 3rd MC (Week 18)	**3.52**	0.90	0.000	**0.60** [1.75−5.29]
Baseline → follow‐up (Week 28)	1.69	0.90	0.061	0.29 [−0.08 to 3.46]
Control group				
Intercept at baseline (Week −244)	**40.10**	1.00	0.000	
Baseline → post 1st MC (Week −242)	0.42	0.89	0.639	0.07 [−1.34 to 2.18]
Baseline → post 2nd MC (Week −190)	**3.82**	0.89	0.000	**0.65** [2.06−5.58]
Baseline → post 3rd MC (Week 18)	**1.80**	0.91	0.049	**0.31** [0.01−3.60]
Baseline → follow‐up (Week 28)	1.59	0.92	0.086	0.27 [−0.23 to 3.41]
Between‐group differences (intervention‐control group)				
Group dif. at baseline	−0.88	1.41	0.534	−0.15 [−3.66 to 1.91]
Group dif. in change after 1st MC	−0.12	1.25	0.922	−0.02 [−2.59 to 2.35]
Group dif. in change after 2nd MC	−0.60	1.25	0.631	−0.10 [−3.07 to 1.87]
Group dif. in change after 3rd MC	1.72	1.28	0.181	0.29 [−0.80 to 4.24]
Group dif. in change at follow‐up	0.10	1.29	0.940	0.02 [−2.44 to 2.63]

*Note*: Significant (*p* < 0.05) estimated values and effect sizes are bolded.

Abbreviations: CI, 95% confidence interval; *d*, Cohen's *d*; dif., difference; ITT, intention to treat.

## DISCUSSION

This RCT examined the longitudinal effects of the MC following responder couples through two or three MCs across more than 5 years. In brief, these couples who had benefitted from two previous MCs showed maintenance of small to medium effects on two out of four outcome measures (MSI and ISQ) at 4 years and 9 months. Hereafter, the changes in couples who received a third MC were not significantly different than the changes in couples who only received the first two MCs. Across the period of the third MC, within‐group analyses revealed that while couples in the current control group showed flat trajectories, couples receiving a third MC experienced small to medium within‐group booster effects on three out of four measures (MSI, CSI, and RAS) with one measure (MSI) maintaining a small to medium effect at the 5‐years‐3‐months follow‐up. Generally, these boosts around the third MC repeat the climbing M pattern of trajectory seen in couples receiving the two previous MCs (Cordova et al., 2014; Trillingsgaard et al., 2016).

Couples receiving a third MC showed small positive anticipation effects on two outcomes, similar to findings from the original RCT. Potential explanations for these anticipation effects could be out‐of‐session mechanisms such as relationship reappraisal, behavioral activation, and motivation from social control and social desirability (see also Trillingsgaard et al., 2016). Cordova (2014) describes how such out‐of‐session mechanisms may be activated already by the reminder of an upcoming MC when partners turn their attention to the relationship with increased care, comparable to the increased brushing and flossing up to a dental checkup. Together with the unexpected small negative anticipation effect in intimacy (though followed by pre‐to‐post improvement), our findings may suggest that couples, when reminded of an upcoming checkup, experience a blend of positive and negative reappraisals of their relationship. Couples may recommit to the relationship in global terms (the positive anticipation effects on MSI and CSI) while also acknowledging some decline in more specific terms (as intimacy).

Generally, we found that the within‐group effects of the third MC were not as large as those of the second MC. This contradicts our initial expectation of *larger* effects among responder couples. Four circumstances of the third MC may help to explain this unexpected finding. First, at the current 4‐years‐9‐months baseline, our responder sample had maintained small to medium effects on half of the measures. This maintenance of effects is rather impressive given the long period of time (4 years) from the second MC to the third MC, and, at the same time, it may leave less room for further improvements. Also, the absence of a boost in intimacy by the third MC may be explained by intimacy theory in that intimate events *gradually* built accept and intimate safety (Cordova, 2014). This nature of intimacy is reflected in its steadier linear growth with more stability across the long period after the second MC in contrast to the decreases found in the climbing M trajectories of the three other measures. This difference in trajectories between intimacy and some measures of relationship satisfaction was also found in previous studies (e.g., Cordova et al., 2014). Second, whereas the first two MCs were scheduled annually, the time space between the second and third MC was much longer (4 years). This time delay may cause couples to retrieve less of their previous learnings reflecting a delayed reinforcement of the booster effects. Third, after the second MC, couples did not expect any further checkups, thus the invitation to a third MC (and to participate in the current study) was unexpected. Effects may be larger when MCs are anticipated and part of a recurrent scheme. Fourth, most couples (*n* = 24; 77%) got a new therapist for the third MC which could have reduced the benefits of an established therapeutic alliance from the first two MC (Hughes et al., 2021). Future studies should test the accumulative effect of recurrent MCs by keeping the therapist and the spacing between MCs consistent.

One measure of relationship satisfaction (MSI) showed generally stronger maintenance of effects than the other measure (CSI), which could be explained by characteristics of the two measures. Item response theory‐based examination of the MSI has found it to discriminate distressed couples particularly well (Balderrama‐Durbin et al., 2015). Specifically, Balderrama‐Durbin et al. (2015) found that respondents should be relatively distressed before they “tipped” from being more likely to report “concerned” than “not concerned” on most of the MSI items (binary scoring of *Yes* or *No*). A sum score of or below 6.00 on the MSI indicates relationship discordance (i.e., four or more areas of concern; Whisman et al., 2009). Couples in the current study exceeded this cut‐point already after the first MC (Week −210 for the intervention group and Week −242 for the control group) and stayed above this cut‐point at all subsequent timepoints, with only one exception for the intervention group (a small drop at Week 0). Thus, small changes in relationship distress among the nondiscording couples in the current study may be better captured by the 6‐ or 7‐point Likert scales of the CSI.

It follows from our selection of a responder sample that our results cannot generalize to the full population. Though the recurring MCs seemed like a beneficial dose and type of help for the current responder couples, this may not generalize to the nonresponder couples. Based on panel plots of the 2‐year trajectories in the 19 nonresponder couples excluded from this study, they were characterized by being either (a) constantly distressed throughout the period with no or close to no response to the two MCs, (b) responding positively to the MCs but quickly dropping down to their baseline, or (c) constantly satisfied. From the perspective of both cost‐effective and ethical delivery of help, the adequacy and necessity of the MC will depend on the individual couple's need and response. For distressed couples who do not respond to the first MC, this intervention is best conceptualized as an important steppingstone toward more intensive interventions tailored to their specific needs.

Finally, findings should be interpreted in light of some limitations. First, this study was short of power to detect statistical significances of small effects, thereby including a risk of a type II error. The current effect sizes (e.g., the between‐group effects of 0.33 and 0.31 found in the MSI) call for future replications with larger samples to detect potential between‐group effects with a power of at least 0.80. As the MC is a brief intervention offered to couples across the continuum of distress, small effects may represent meaningful benefits in a public health perspective. Second, our design did not allow us to attribute any of the effects to mechanisms of change specifically linked to the MC. Studies comparing the MC to another intervention or studies specifically designed to disentangle mechanisms of change are needed for that purpose.

## CONCLUSION

In conclusion, this study indicates that couples who benefit from two annual MCs can maintain some of these improvements in relationship health across long periods of time, while the ability to boost such improvements with a third MC was not clearly supported. Thus, improvements for couples assigned to a third MC were not statistically greater than the longitudinal effects seen for control couples who similarly benefitted from two MCs (between‐group effects). Our results could, however, point to the importance of further examination of the longitudinal booster effects of regular MCs in a larger sample, as the current study did not have power to detect small between group effect sizes. The average size of effects achieved from the MC model may not point to this intervention as a stand‐alone treatment for chronic relationship distress, but the current results point to the MC as a relevant and tolerable intervention that support couples in sustaining relationship functioning across longer periods of time. As such, the MC model should be considered as an integrated part of a more comprehensive public health strategy for preventing couple distress in the future.

## ENDNOTES

1 A free and revised 10‐item version of the RAS is available online: https://psy.au.dk/en/the-couple-and-family-lab/materials-for-researchers-and-clinicians

2 The maximum possible number of observations = (32 couples in intervention group x 4 timepoints x 4 outcome variables) + (31 couples in control group x 3 timepoints x 4 outcome variables) = 884.

## Appendix

**Table A1:** Descriptive statistics for outcome variables across 5 years and 3 months, mean (*SD*)

Variable group	Week −244	Week −242	Week −223	Week −210	Week −197	Week −190	Week −140	Week 0	Week 16	Week 18	Week 28
**Marital satisfaction inventory‐B**											
Current intervent.	5.30 (1.49)	5.97 (1.81)	5.92 (2.02)	6.17 (1.90)	6.25 (1.69)	7.02 (1.82)	6.42 (1.98)	5.95 (2.09)	6.34 (2.23)	6.68 (2.06)	6.62 (2.10)
Current control	5.39 (1.68)	6.52 (1.74)	6.73 (1.77)	6.58 (1.94)	6.63 (1.75)	7.35 (1.46)	6.40 (1.70)	6.34 (1.27)	‐	6.69 (1.50)	6.62 (1.66)
Nonresponder	5.37 (2.60)	5.69 (2.54)	5.26 (2.82)	5.28 (3.02)	5.18 (2.39)	4.87 (3.11)	5.17 (3.37)	‐	‐	‐	‐
Original control	5.04 (2.07)	5.33 (2.29)	‐	‐	‐	5.41 (2.26)	5.48 (2.06)	‐	‐	‐	‐
**Couple satisfaction index‐16**											
Current intervent.	58.98 (9.05)	59.50 (10.02)	58.30 (10.97)	59.13 (11.53)	60.56 (11.61)	62.83 (9.11)	60.32 (10.54)	58.27 (11.74)	60.58 (13.22)	60.53 (11.50)	59.22 (11.88)
Current control	59.71 (10.75)	63.61 (8.47)	60.95 (11.5)	60.32 (10.52)	63.92 (8.00)	66.87 (6.16)	62.33 (8.6)	60.48 (9.87)	‐	61.31 (9.78)	61.71 (9.25)
Nonresponder	56.71 (14.36)	56.78 (14.27)	53.08 (16.64)	52.56 (18.82)	55.34 (12.75)	49.63 (19.63)	49.40 (20.03)	‐	‐	‐	‐
Original control	54.46 (14.2)	53.99 (14.07)	‐	‐	‐	55.40 (15.10)	56.41 (13.65)	‐	‐	‐	‐
**Intimate safety questionnaire**											
Current intervent.	3.14 (0.30)	3.14 (0.32)	3.11 (0.32)	3.18 (0.32)	3.19 (0.35)	3.28 (0.33)	3.21 (0.33)	3.24 (0.38)	3.15 (0.41)	3.20 (0.40)	3.17 (0.47)
Current control	3.25 (0.26)	3.28 (0.28)	3.23 (0.35)	3.28 (0.31)	3.31 (0.30)	3.39 (0.24)	3.34 (0.34)	3.32 (0.29)	‐	3.31 (0.35)	3.28 (0.33)
Nonresponder	3.16 (0.28)	3.13 (0.35)	3.13 (0.46)	3.02 (0.60)	3.18 (0.36)	2.99 (0.56)	3.00 (0.63)	‐	‐	‐	‐
Original control	3.11 (0.42)	3.11 (0.45)	‐	‐	‐	3.12 (0.43)	3.18 (0.40)	‐	‐	‐	‐
**Responsive and attention scale**											
Current intervent.	39.22 (5.06)	39.52 (5.35)	41.06 (5.21)	40.39 (5.34)	41.56 (6.33)	42.44 (5.13)	41.73 (5.77)	40.72 (6.24)	40.02 (5.97)	42.62 (5.57)	40.78 (5.46)
Current control	40.10 (6.61)	40.52 (5.37)	40.71 (7.36)	41.15 (6.78)	42.52 (5.23)	43.92 (4.58)	39.90 (5.65)	41.35 (6.97)	‐	41.95 (6.81)	41.64 (5.89)
Nonresponder	37.61 (6.89)	38.56 (6.34)	36.55 (6.91)	35.58 (8.99)	39.26 (6.49)	36.50 (7.97)	38.07 (8.11)	‐	‐	‐	‐
Original control	37.80 (7.17)	37.52 (7.40)	‐	‐	‐	37.49 (7.91)	38.10 (8.37)	‐	‐	‐	‐
**Number of couples** a											
Current intervent.	32/31	32/32	32/32	32/32	32/32	32/32	31/31	32/32	31/31	30/30	30/30
Current control	31/31	31/31	28/28	31/31	31/31	31/31	29/29	31/31	‐	29/29	28/28
Nonresponder	19/17	18/18	19/18	18/18	19/19	19/19	15/15	‐	‐	‐	‐
Original control	117/114	112/112	‐	‐	‐	100/99	83/83	‐	‐	‐	‐

Abbreviations: CSI, couple satisfaction index; Intervent., intervention; MSI, marital satisfaction inventory—brief; RAS, responsive attention scale.

^a^ Left to the slash is *N* of CSI, MSI, and RAS. Right to the slash is *N* of ISQ.
